# Augmenter of Liver Regeneration-Modified Adipose Mesenchymal Stem Cell-Derived Exosomes Repairs Liver Damage by Regulating Endoplasmic Reticulum Stress and Pyroptosis in a *Minipig* Model of Liver Injury

**DOI:** 10.3390/antiox15040450

**Published:** 2026-04-03

**Authors:** Yajun Ma, Tao Liu, Lei Cao, Pujun Li, Xiangyu Lu, Yue Wang, Hongbin Wang

**Affiliations:** 1 College of Veterinary Medicine, Northeast Agricultural University, Harbin 150030, China; b210601014@neau.edu.cn (Y.M.); liutao2015@neau.edu.cn (T.L.); caolei@neau.edu.cn (L.C.); pujunli@neau.edu.cn (P.L.); b220601009@neau.edu.cn (X.L.); 2 College of Animal Science and Technology, Henan University of Science and Technology, Luoyang 471023, China; wangyue629@haust.edu.cn

**Keywords:** ADSC-ALR-Exo, hepatic IRI, minipig, endoplasmic reticulum stress, pyroptosis

## Abstract

Adipose mesenchymal stem cell-derived exosomes (ADSC-Exo) have demonstrated therapeutic effects in liver diseases and injuries. The Augmenter of Liver Regeneration (ALR), a novel hepatic trophic growth factor, promotes hepatic structural and functional recovery. In this study, we constructed ALR-overexpressing ADSC-Exo (ADSC-ALR-Exo) by harnessing the messaging capacity of ADSC-Exo, and analyzed the effects of ADSC-ALR-Exo on hepatic ischemia–reperfusion injury (IRI) combined with partial hepatectomy in a *minipig* model. Our results indicated that, compared to the ADSC-Exo group, the ADSC-ALR-Exo group exhibited a significant reduction in reactive oxygen species (ROS) levels, alongside a notable increase in the activity of antioxidant enzymes superoxide dismutase (SOD) and catalase (CAT). Furthermore, there was a marked decrease in malondialdehyde (MDA) content. Concurrently, the concentrations of pro-inflammatory factors in the blood (IL-1β, IL-18, and TNF-α) and liver tissue (IL-1β, IL-18, IL-6, and TNF-α) were significantly lower in the ADSC-ALR-Exo group, while the level of the anti-inflammatory factor IL-10 in the blood was significantly elevated. Additionally, ALR enrichment enhanced the inhibitory effect of ADSC-ALR-Exo on endoplasmic reticulum stress-related pathways, specifically ATF6, IRE1α, and PERK. Compared to ADSC-Exo, the ADSC-ALR-Exo intervention was also more effective in reducing the expression levels of NLRP3, caspase-1, and GSDMD, thereby decreasing the incidence of pyroptosis. In conclusion, ADSC-ALR-Exo mitigated liver injury by inhibiting endoplasmic reticulum stress and cellular pyroptosis induced by liver injury.

## 1. Introduction

Hepatic ischemia–reperfusion injury (IRI) is a common and severe complication encountered in clinical scenarios such as liver surgery, organ transplantation, and shock reperfusion. Its pathogenesis is complex, involving multiple pathological processes, including oxidative stress, inflammatory response, calcium homeostasis disruption, endoplasmic reticulum (ER) stress, and pyroptosis [1]. In recent years, the interaction between ER stress and pyroptosis in hepatic IRI has garnered increasing attention. When hepatic blood flow is restricted, the insufficient oxygen and nutrient supply within hepatocytes disrupts cellular metabolism, leading to the accumulation of large quantities of unfolded or misfolded proteins. The aggregation of these abnormal proteins directly activates the ER stress pathway. ER stress mediates the release of cytokines such as tumor necrosis factor-alpha (TNF-α) and interleukin-6 (IL-6), which exacerbate local inflammation and further damage hepatocytes [2,3]. Additionally, ER stress may amplify inflammatory responses by activating immune cells such as macrophages [4]. The ER serves as the primary site for calcium (Ca^2+^) storage and release; therefore, ER stress can disrupt intracellular Ca^2+^ homeostasis. This disruption leads to a continuous influx of Ca^2+^ from the ER into the mitochondria, which induces oxidative damage [5]. Research conducted on a fatty liver IRI model has demonstrated that ER stress promotes mitochondrial calcium overload in macrophages. This overload, in turn, stimulates the production of reactive oxygen species (ROS), which further activates the ROS/NLRP3 signaling pathway, leading to pyroptosis and exacerbating hepatic IRI [6]. The ER stress-related pathways, including inositol-requiring enzyme 1 (IRE1) and protein kinase RNA-like endoplasmic reticulum kinase (PERK), via the CHOP-TXNIP axis, not only mediate apoptotic signals but also influence the activation of the NOD-like receptor thermal protein domain-associated protein 3 (NLRP3) inflammasome [7]. Research indicates that in certain liver disease models, persistent ER stress is closely associated with hepatocyte death, potentially compromising the liver’s regenerative capacity [8]. Concurrently, microenvironmental alterations induced by ER stress may disrupt the function of other cell types, thereby affecting overall liver remodeling.

Mesenchymal stem cells (MSCs) are a widely available type of adult stem cell known for their self-renewal and multipotent differentiation potential. They can secrete various bioactive molecules, demonstrating significant therapeutic potential in regenerative medicine and immune-mediated inflammatory diseases [9]. However, challenges related to the safety, immunogenicity, and in vivo survival rates of direct MSCs transplantation have prompted researchers to explore the role of MSC-derived exosomes (MSC-Exo) [10,11]. MSC-Exo facilitates the delivery of bioactive molecules via mechanisms such as membrane fusion with recipient cells, receptor-mediated endocytosis, or activation of surface receptors, thereby promoting tissue regeneration and repair [12]. Furthermore, MSC-Exo influences both innate and adaptive immune responses through various pathways. On one hand, exosomes can suppress the expression of pro-inflammatory cytokines, such as TNF-α and interleukin-1β (IL-1β), while promoting the secretion of anti-inflammatory factors like interleukin-10 (IL-10) and transforming growth factor-β (TGF-β), which helps inhibit excessive inflammatory responses [13]. On the other hand, miRNAs and proteins contained within exosomes can regulate the phenotype and function of immune cells. For instance, they promote M2 polarization of macrophages [14], inhibit dendritic cell maturation [15], and reduce T cell proliferation [16]. Collectively, these effects support the restoration of immune homeostasis and alleviate inflammation-related diseases. Additionally, MSC-Exo can mitigate cellular oxidative damage by delivering antioxidant molecules and regulating relevant signaling pathways [17]. Antioxidant-related miRNAs contained in exosomes can reduce ROS levels, alleviate endoplasmic reticulum stress, and inhibit apoptosis pathways, thereby protecting tissues from oxidative stress-related damage [18,19]. In the liver IRI model, MSC-Exo has demonstrated the potential to mitigate oxidative damage, promote cell survival, and restore functional recovery [20,21].

MSC-Exo, as small extracellular vesicles, are capable of carrying bioactive molecules, including proteins, lipids, mRNA, miRNA, and other noncoding RNAs. Compared to mesenchymal stem cells (MSCs), MSC-Exo exhibit lower immunogenicity and enhanced chemical stability, suggesting their potential as delivery vehicles for transferring specific drugs or targeted genes/proteins to other cells [22,23]. Reports indicate that utilizing MSC-Exo to deliver microRNA-124 (miR-124), known for its tumor-suppressive properties, along with the programmed cell death protein 1 (PD-1) gene into glioblastoma, resulted in the MSC-Exo/miR-124-PD-1 complex effectively inducing apoptosis in GBM cells, significantly inhibiting tumor growth, and activating immune cells (T cells and dendritic cells) while reducing immunosuppressive cell populations [24]. Zhang and colleagues similarly reported that, compared to unmodified MSC-Exo, MSC-Exo engineered with miR-486-5p promoted the proliferation and migration of irradiated MLE-12 cells, rescued cells from ferroptosis, suppressed the expression of fibrotic genes in MLE-12 cells, and alleviated inflammation and pulmonary fibrosis in mice with radiation-induced lung injury [25]. Guo et al. demonstrated that IL-35-modified adipose-derived stem cells (ADSCs) effectively suppress acute rejection after cardiac transplantation, with their derived exosomes serving as the primary effector molecules [26]. Sun et al. also reported that HIF-1α-overexpressed MSC-Exos more effectively promote angiogenesis and inhibit fibrosis following myocardial infarction injury, thereby protecting cardiac function [27]. Furthermore, in a unilateral ureteral obstruction *mouse* model, GDNF-modified MSC-Exos significantly reduced peritubular capillary loss in renal tubulointerstitial fibrosis. In vitro, they similarly promoted migration and angiogenesis in human umbilical vein endothelial cells following hypoxia/serum deprivation injury while reducing cell death [28]. These reports indicate that modifying and engineering MSC-Exo using genetic engineering techniques endows them with more targeted therapeutic effects. These genetically modified MSC-Exo demonstrate broad application prospects in the biomedical field. However, there have been few reports to date on gene-modified MSC-Exo for treating liver injury. Therefore, we utilized ALR (a novel hepatic trophic growth factor) to genetically modify ADSC-derived exosomes (ADSC-Exo) to obtain ADSC-Exo with high ALR expression (ADSC-ALR-Exo), implanted them into the liver, and investigated whether ADSC-ALR-Exo has improved therapeutic effects on liver injury compared to ADSC-Exo.

In this study, we investigated whether ADSC-ALR-Exo exhibit stronger anti-inflammatory and antioxidant effects compared to those secreted by unmodified ADSC-Exo in a *minipig* laparoscopic liver IRI model combined with partial hepatectomy. Additionally, we observed the effects of ADSC-ALR-Exo on intervening in ER stress and pyroptosis induced by liver injury.

## 2. Materials and Methods

### 2.1. Minipig

Twenty-four Guangxi Bama miniature *pigs*, 4–6 months old, 20–25 kg, half male and half female, were reared under uniform management conditions and used in the experimental study after both clinical and laboratory physical examinations showed good health. The animal experiments involved in this study were approved by the Animal Ethics Committee of Northeast Agricultural University (No. NEAUEC2023.03.132).

### 2.2. Isolation and ALR Overexpression Modification of ADSCs (ALR-ADSCs)

Fat blocks at the groin of *minipigs* were isolated under aseptic conditions, and ADSCs were obtained using enzymatic digestion [29]. ADSCs were cultured in low-glucose Dulbecco’s modified Eagle medium (Gibco, Carlsbad, CA, USA) supplemented with 10% FBS (Clark, McLean, VA, USA), 2 mM L-glutamine, and 100 μg/mL penicillin and streptomycin (Solarbio, Beijing, China). The acquisition method for ALR-ADSCs can be found in our reported article [30].

### 2.3. Isolation of Exosomes

Cell culture supernatants for exosome isolation were substituted with starvation medium (DMEM medium with 1% glutamine and 1% penicillin) 36 h before exosome isolation. Exosomes were extracted using differential ultracentrifugation [31].

### 2.4. Establishment of Laparoscopic Hepatic IRI Combined with Partial Hepatectomy in Minipigs

The operating room is preemptively disinfected using ultraviolet light. Laparoscopic instruments are sterilized with a low-temperature hydrogen peroxide plasma sterilizer, while conventional surgical instruments and supplies are sterilized using high-pressure steam. Prior to surgery, the health status of the *minipigs* is confirmed through clinical and laboratory examinations. The surgical site is shaved, cleaned, and disinfected. An intramuscular injection of atropine sulfate (0.05 mg/kg) is administered. Fifteen minutes later, Zoletil^®^ 100 is administered at a dosage of 1 mg/kg via the marginal ear vein, followed by a slow injection of Propofol until successful induction of anesthesia in the *minipig* is achieved, as indicated by jaw relaxation, tongue protrusion without retraction, and the absence of eyelid reflex. Tracheal intubation is then performed, followed by the connection of the ventilator, anesthesia machine, and monitoring equipment. The concentration of the inhalation anesthetic is set at 2–3% (Isoflurane). During the surgical procedure, continuous monitoring of the *minipig*’s body temperature, respiratory rate, heart rate, blood pressure, and other parameters is conducted. These measurements are combined with observations of the *minipig*’s intraoperative status to assess surgical progress and anesthetic conditions. The liver injury model was achieved by treatment with Ischemia–reperfusion of the right lobe and resection of the left lobe according to our previous study [29]. The animals were randomly divided into the Sham group, IRI group, ADSC-Exo group, and ADSC-ALR-Exo group (n = 6 per group). The Sham group only maintained the same pneumoperitoneum and anesthesia. The IRI group, ADSC-Exo group, and ADSC-ALR-Exo group were injected with 5 mL PBS, ADSC-Exo (5 × 10^9^ ADSC-Exo/kg), and ADSC-ALR-Exo (5 × 10^9^ ADSC-ALR-Exo/kg) through the portal vein after the operation. Blood and liver tissue samples were collected preoperatively and at 1, 3, and 7 days postoperatively.

### 2.5. Antioxidant Enzyme Activities and Analysis of Lipid Peroxidation

Catalase (CAT), glutathione peroxidase (GSH-Px), and superoxide dismutase (SOD) activities, and malondialdehyde (MDA) were measured using specific assay kits according to the manufacturer’s instructions (Nanjing Jiancheng, Nanjing, China).

### 2.6. ELISA

IL-1β, IL-6, IL-10, interleukin 18 (IL-18), and TNF-α were measured using specific ELISA kits according to the manufacturer’s instructions (Solarbio, China).

### 2.7. Ultrastructure Observation

The morphology of liver tissues was detected by transmission electron microscopy (Hitachi H-7650, Tokyo, Japan). Fresh liver tissue samples were sequentially fixed with 2.5% glutaraldehyde solution, embedded, cut into ultrathin sections, and then observed under a transmission electron microscope.

### 2.8. Immunofluorescence Staining

Paraffin sections were dewaxed, rehydrated, and antigen-repaired. The sections were blocked with bovine serum albumin (BSA) and incubated with a primary antibody specific to GSDMD (Proteintech, Rosemont, IL, USA, 1:200), followed by a fluorescent secondary antibody (ABclonal, Wuhan, China, 1:500). All images were obtained by a fluorescent microscope (Leica, Wetzlar, Germany).

### 2.9. DHE Staining

Fresh liver tissue was OCT-embedded, snap-frozen, sectioned, and DHE-stained according to the manufacturer’s instructions (Thermo Fisher Scientific, Waltham, MA, USA). The sections were observed and recorded under a fluorescence microscope (Leica, Germany).

### 2.10. Western Blotting

Total protein was extracted from liver tissue samples using RIPA protein lysate (Beyotime, Shanghai, China). Total protein concentration was detected and quantified using a BCA kit (Beyotime, China). Protein samples were loaded onto an SDS-PAGE gel for separation, then transferred to a nitrocellulose membrane (Millipore, Burlington, MA, USA). The blots were blocked with 5% nonfat dry milk in TBST for 2 h at room temperature and then incubated overnight at 4 °C with primary antibodies against GRP78 (Wanlei, Shenyang, China, 1:1500), ATF6α (Abcam, Cambridge, UK, 1:1000), p-eIF2α (Abcam, UK, 1:5000), eIF2α (Cell Signaling Technolioy, Danvers, MA, USA, 1:1000), ATF4 (Santa Cruz, CA, USA, 1:500), XBP1s (Santa Cruz, USA, 1:500), β-actin (Proteintech, USA, 1:4000), and α-tubulin (Proteintech, USA, 1:5000). After washing with TBST, the blots were incubated with HRP-Goat-Anti-Rabbit secondary antibody (Proteintech, USA, 1:10,000), and HRP-Goat-Anti-*Mouse* secondary antibody (Proteintech, USA, 1:5000) for 1 h at room temperature. Positive bands were detected using an enhanced ECL reagent (Meilunbio, Dalian, China) on an AI600 System (GE Healthcare, Pollards Wood, UK) and quantified by ImageJ software (1.53, NIH, Bethesda, MD, USA).

### 2.11. Quantitative Real-Time PCR Assays

Total RNA was extracted from liver tissue using TRIzol lysate (Invitrogen, Carlsbad, CA, USA), and reverse transcribed into cDNA according to the manufacturer’s instructions. Quantitative PCR was conducted using SYBR Green I fluorescent dye (Vazyme, Nanjing, China) on a Roche LightCycler 480 (Roche, Basel, Switzerland). Relative mRNA expression levels were calculated using the 2^−ΔΔCT^ method and were normalized to the corresponding expression levels of β-actin. The primer sequences used to amplify the *pig* RNA are shown in Table 1.

### 2.12. Statistical Analysis

All data were expressed as the mean ± SD and compared using one-way ANOVA in SPSS Statistics 27. A *p*-value < 0.05 is considered statistically significant.

## 3. Results

### 3.1. ADSC-ALR-Exo Mitigates Ultrastructural Damage to Hepatocytes Caused by Liver Injury

**Figure 1:**
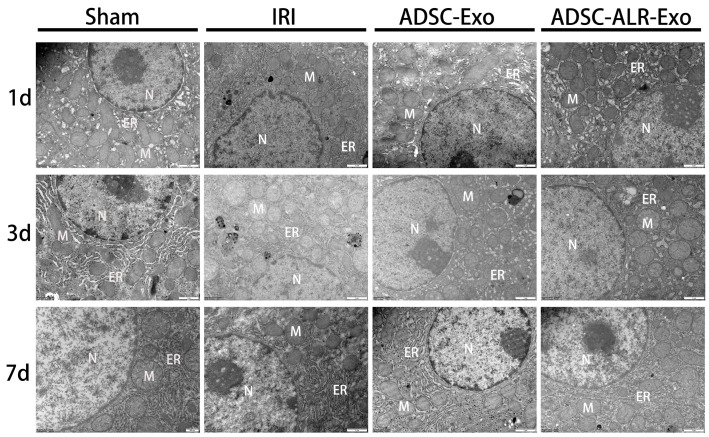
ADSC-ALR-Exo mitigates ultrastructural damage to hepatocytes caused by liver injury. Scale bar: 1 μm. N represents the nucleus; ER represents the endoplasmic reticulum; M represents mitochondria.

As shown in Figure 1, the sham group exhibited normal histological structures without significant damage at any time point. Following hepatic IRI combined with partial hepatectomy, varying degrees of injury were observed across all surgical groups, with the most severe damage occurring at 1 and 3 days post-surgery. On the first postoperative day, hepatocytes in the IRI group displayed severe nuclear membrane shrinkage and even rupture. The cytoplasmic structures were disorganized, organelles were dispersed, the endoplasmic reticulum was swollen, and mitochondria exhibited irregular morphology with blurred, swollen structures; some mitochondria were even absent. Hepatocyte damage in both the ADSC-Exo and ADSC-ALR-Exo groups was milder compared to the IRI group. Autophagic structures were observed within hepatocytes across all surgical groups. By postoperative day 3, hepatic cell damage had diminished in all groups, accompanied by a corresponding reduction in autophagic structures. However, the IRI group still exhibited significant lesions in nuclei, cytoplasm, and organelles. By postoperative day 7, damage in all groups had largely normalized. These findings suggest that ADSC-ALR-Exo mitigates morphological alterations in hepatocytes and may contribute to the repair of damage to hepatocytes, mitochondria, and the endoplasmic reticulum.

### 3.2. ADSC-ALR-Exo Reduces Oxidative Stress Induced by Liver Injury

**Figure 2:**
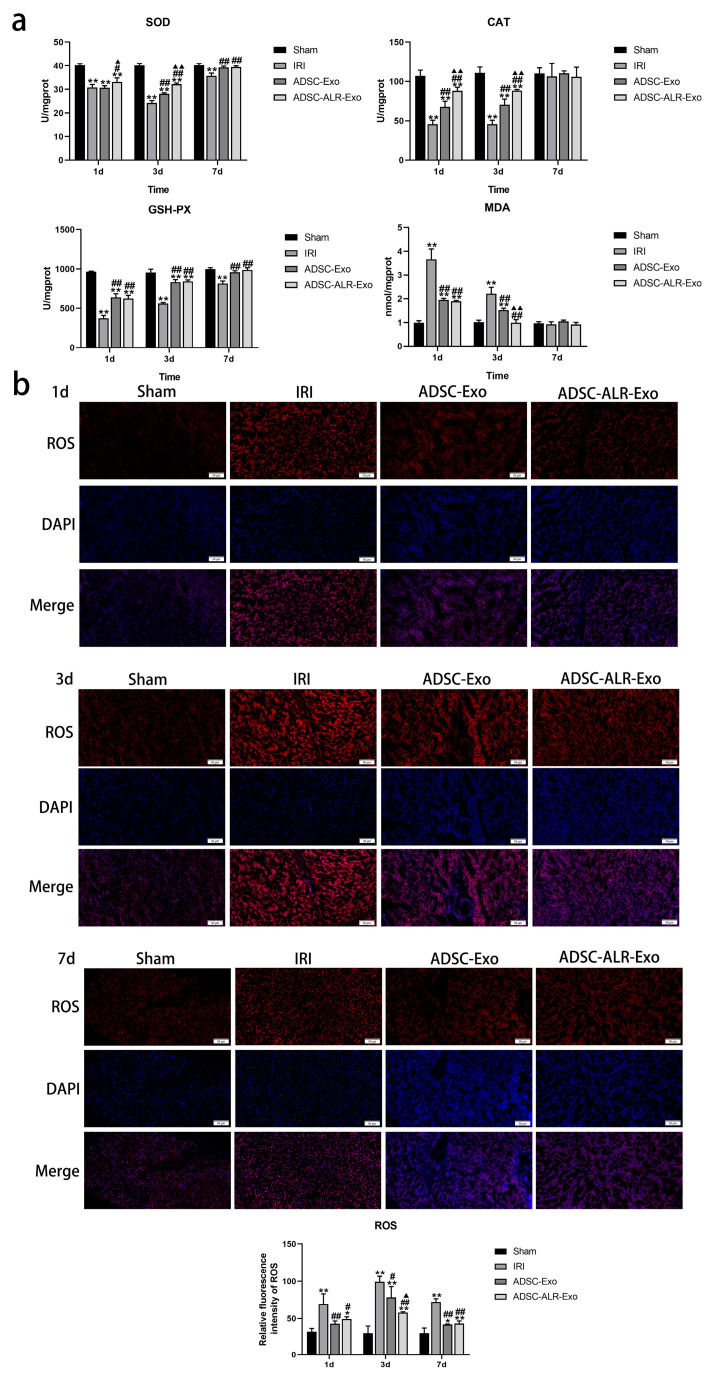
ADSC-ALR-Exo reduces oxidative stress induced by liver injury. (**a**) Biochemical level detection of oxidative stress-related markers in liver tissue. (**b**) Observation of ROS levels in liver tissue. Scale bar: 50 µm. Quantification of the fraction of ROS using ImageJ. The results are presented as the means ± standard deviations (SDs). * *p* < 0.05, ** *p* < 0.01, compared with the Sham group. # *p* < 0.05, ## *p* < 0.01, compared with the IRI group. ▲ *p* < 0.05, ▲▲ *p* < 0.01, relative to the ADSC-Exo group.

During hepatic IRI, a significant amount of ROS is released, initiating a series of oxidative stress-related chain reactions that lead to cellular damage and liver dysfunction [32,33]. MDA is a marker of lipid peroxidation, with its concentration indicating the extent of oxidative damage in the body [34]. SOD, CAT, and GSH-PX are key antioxidant enzymes that participate in the oxidative stress response. Dysregulation of these essential antioxidant enzymes plays a crucial role in the pathogenesis of various diseases associated with oxidative stress-induced damage [35,36]. As illustrated in Figure 2a, we assessed the activities of the antioxidant enzymes SOD, CAT, and GSH-PX, along with the levels of the lipid peroxidation product MDA, in liver tissue following liver injury. The results demonstrated that the activities of SOD, CAT, and GSH-PX significantly decreased (*p* < 0.01) across all surgical groups at 1 day and 3 days post-establishment of the IRI combined with partial liver resection model. In comparison to the IRI group, the reduction in antioxidant enzyme activity was relatively less pronounced in both intervention groups, with ADSC-ALR-Exo exhibiting a more significant effect on SOD and CAT (*p* < 0.05). Correspondingly, MDA levels in liver tissue increased in all surgical groups at 1 day post-surgery (*p* < 0.01), with a comparatively smaller increase observed in the two intervention groups. By postoperative day 3, MDA levels decreased in all surgical groups, with the ADSC-ALR-Exo group returning to sham levels. Unlike the other antioxidant enzymes, GSH-PX levels did not show significant differences between the ADSC-Exo and ADSC-ALR-Exo groups. We hypothesize that this may be due to the timing of sample collection, which could have missed earlier or later changes in GSH-PX levels. Alternatively, it is possible that the enrichment of ALR does not enhance the effect of ADSC-Exo on GSH-PX. On postoperative day 7, aside from SOD and GSH-PX levels remaining significantly lower in the IRI group compared to the sham group (*p* < 0.01), no significant differences were noted in other measured parameters among the groups (*p* > 0.05). To further investigate the protective effect of ADSC-ALR-Exos on oxidative stress injury, we observed DHE staining in liver tissue after injury. The results (Figure 2b) revealed that after hepatic IRI combined with partial hepatectomy injury, the intensity of DHE fluorescence in liver tissue was significantly increased, and many ROSs were produced in each surgical group. Compared with the IRI group, DHE fluorescence intensity in liver tissues was decreased (*p* < 0.05), and ROS content was reduced after ADSC-Exo or ADSC-ALR-Exo intervention. A significantly lower (*p* < 0.05) ROS content in liver tissue from the ADSC-ALR-Exo group compared with the ADSC-Exo group was observed at 3 days post-surgery. These findings suggest that ADSC-ALR-Exo effectively reduces lipid peroxidation products and ROS in liver tissue and enhances antioxidant enzyme activity, demonstrating notable advantages in alleviating oxidative stress damage.

### 3.3. ADSC-ALR-Exo Reduces the Inflammatory Response Induced by Liver Injury

**Figure 3:**
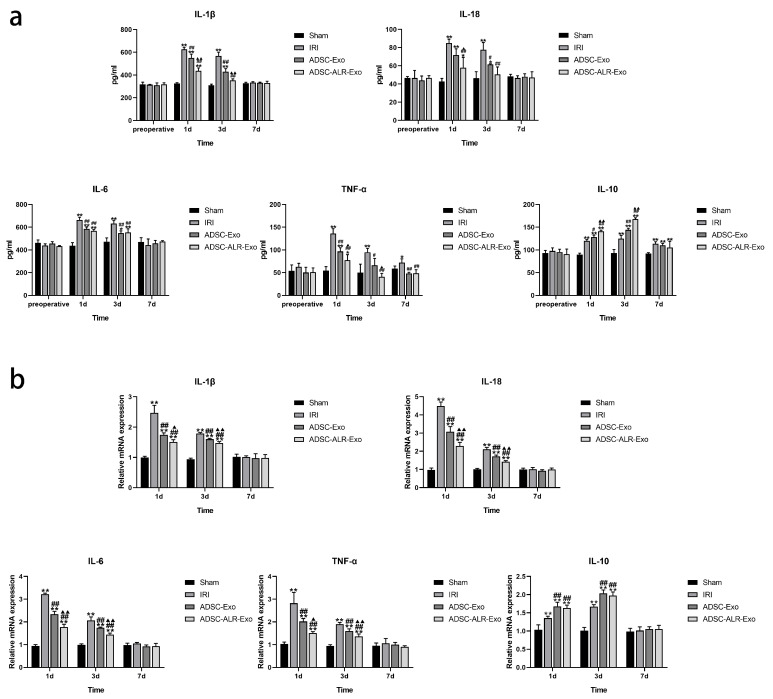
ADSC-ALR-Exo reduces the inflammatory response induced by liver injury. (**a**) Serum concentrations of factors associated with inflammation. (**b**) Expression levels of mRNAs related to inflammation in hepatic tissue. The results are presented as the means ± standard deviations (SDs). * *p* < 0.05, ** *p* < 0.01, compared with the Sham group. # *p* < 0.05, ## *p* < 0.01, compared with the IRI group. ▲ *p* < 0.05, ▲▲ *p* < 0.01, relative to the ADSC-Exo group.

Hepatic IRI initiates an inflammatory response that leads to the release of significant quantities of inflammatory mediators. These mediators promote the infiltration of inflammatory cells and contribute to hepatocyte death [32,33]. Therefore, effective regulation of the inflammatory response represents a crucial target for the prevention and treatment of hepatic IRI. In this context, we investigated the changes in the concentrations of various inflammation-related factors in serum and liver tissue following hepatic IRI combined with partial hepatectomy. The results indicated (Figure 3a) that one day post-surgery, serum levels of pro-inflammatory factors IL-1β, IL-18, IL-6, TNF-α, and the anti-inflammatory factor IL-10 were significantly elevated in the IRI, ADSC-Exo, and ADSC-ALR-Exo groups compared to the Sham group (*p* < 0.05). Interventions with ADSC-Exo and ADSC-ALR-Exo significantly reduced the secretion of pro-inflammatory factors while enhancing the secretion of anti-inflammatory factors, with ADSC-ALR-Exo demonstrating a more pronounced therapeutic effect. By postoperative day 3, pro-inflammatory factor concentrations decreased, and anti-inflammatory factor concentrations increased across all surgical groups. Notably, serum levels of IL-1β, IL-18, and TNF-α in the ADSC-ALR-Exo group did not differ significantly from those in the Sham group (*p* > 0.05). By postoperative day 7, serum levels of IL-1β, IL-18, and IL-6 had normalized in all surgical groups (*p* > 0.05), while IL-10 levels remained elevated (*p* < 0.01). The TNF-α concentration in the IRI group serum remained significantly higher than that of the Sham group (*p* < 0.05). In contrast, both intervention groups exhibited serum TNF-α levels restored to those of the Sham group (*p* > 0.05). Changes in mRNA levels of inflammatory-related factors in liver tissue (Figure 3b) indicated that at 1 day and 3 days post-surgery, the mRNA levels of pro-inflammatory factors *IL-1β*,* IL-18*,* IL-6*,* TNF-α*, and the anti-inflammatory factor *IL-10* were significantly elevated in the liver tissue of all surgical groups compared to the Sham group (*p* < 0.01). In comparison to the IRI group, both intervention groups exhibited significantly reduced mRNA levels of pro-inflammatory factors (*p* < 0.01) and significantly elevated mRNA levels of the anti-inflammatory factor *IL-10* (*p* < 0.01) in liver tissue. Furthermore, the ADSC-ALR-Exo group demonstrated a more pronounced reduction in mRNA levels of pro-inflammatory factors compared to the ADSC-Exo group (*p* < 0.05). We observed that, in comparison to the ADSC-Exo group, the trends in IL-6 and IL-10 levels in both blood and liver tissue were not entirely consistent within the ADSC-ALR-Exo group. We hypothesize that this inconsistency may be attributed to insufficient precision in our sampling time points, which could have hindered our ability to capture the optimal intervention peaks of ADSC-ALR-Exo on IL-6 and IL-10. Additionally, it is plausible that following portal vein injection, ADSC-ALR-Exo is distributed not only to liver tissue but also throughout the body, thereby stimulating various liver cells as well as circulating immune cells and cells in other organs. This widespread distribution may contribute to the observed differences in the release of IL-6 and IL-10 in liver tissue and blood. By postoperative day 7, no significant differences were observed among the groups (*p* > 0.05). These findings suggest that ADSC-ALR-Exo effectively suppresses the secretion of pro-inflammatory factors while promoting the secretion of anti-inflammatory factors, indicating a distinct advantage in mitigating inflammatory responses.

### 3.4. ADSC-ALR-Exo Reduces ER Stress Induced by Liver Injury

**Figure 4:**
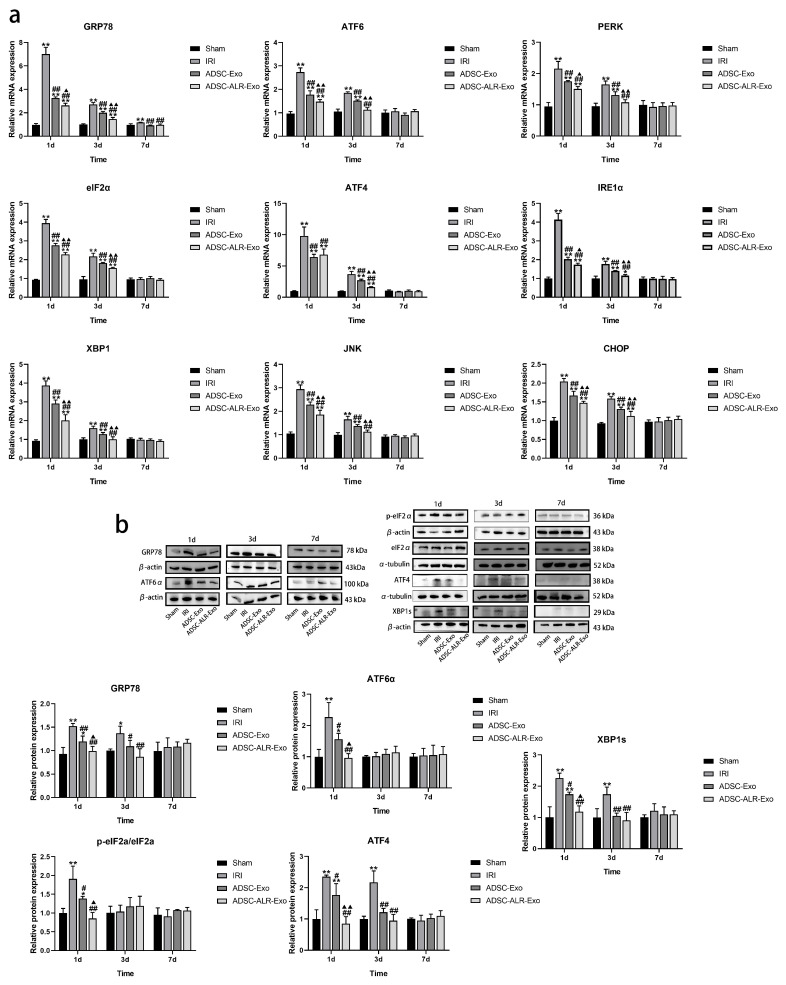
ADSC-ALR-Exo reduces ER stress induced by liver injury. (**a**) Expression levels of mRNAs related to indicators of ER stress in the liver. (**b**) Protein expression levels of ER stress indicators in the liver. The results are presented as the means ± standard deviations (SDs). * *p* < 0.05, ** *p* < 0.01, compared with the Sham group. # *p* < 0.05, ## *p* < 0.01, compared with the IRI group. ▲ *p* < 0.05, ▲▲ *p* < 0.01, relative to the ADSC-Exo group.

ER stress is recognized as a key pathological mechanism in various diseases [37]. Persistent or excessive ER stress can lead to cellular dysfunction and apoptosis. It primarily regulates intracellular stress responses through three signaling pathways—PERK, IRE1, and ATF6—to maintain cellular homeostasis [38]. Our findings (Figure 4a) demonstrate that, at 1 day post-surgery, the IRI group, ADSC-Exo group, and ADSC-ALR-Exo group exhibited significantly increased expression levels of ER stress-related genes *GRP78*, *ATF6*, *PERK*, *eIF2α*, *ATF4*, *IRE1α*, *XBP1*, *JNK*, and *CHOP* compared to the Sham group (*p* < 0.01). Among these groups, both the ADSC-Exo and ADSC-ALR-Exo groups showed significantly lower mRNA expression levels for all detected markers compared to the IRI group (*p* < 0.01). Furthermore, in the ADSC-ALR-Exo group, mRNA expression levels for all markers, except *ATF4*, were significantly lower than those in the ADSC-Exo group (*p* < 0.05). At 3 days post-surgery, mRNA expression levels of relevant markers decreased across all surgical groups. The IRI and ADSC-Exo groups maintained elevated expression levels (*p* < 0.01), while in the ADSC-ALR-Exo group, only *GRP78*, *eIF2α*, *ATF4*, *IRE1α*, and *CHOP* remained significantly higher than the Sham group (*p* < 0.05), while other indicators had returned to normal levels (*p* > 0.05). By postoperative day 7, *GRP78* mRNA expression in the IRI group remained significantly elevated compared to the Sham group (*p* < 0.01), while no significant differences were observed among other factors across the groups (*p* > 0.05). Furthermore, we assessed the expression levels of endoplasmic reticulum stress-related proteins at various time points (Figure 4b). The results aligned with the gene expression data: one day post-surgery, protein expression levels of GRP78, ATF6α, p-eIF2α/eIF2α, ATF4, and XBP1s in the IRI group were significantly higher than those in the Sham group (*p* < 0.01). Both intervention groups showed significantly lower expression levels compared to the IRI group (*p* < 0.05), with ADSC-ALR-Exo demonstrating a more pronounced reduction trend (*p* < 0.05). These findings suggest that ADSC-ALR-Exo effectively inhibits the ATF6, IRE1α, and PERK pathways associated with endoplasmic reticulum stress.

### 3.5. ADSC-ALR-Exo Reduces Pyroptosis Induced by Liver Injury

**Figure 5:**
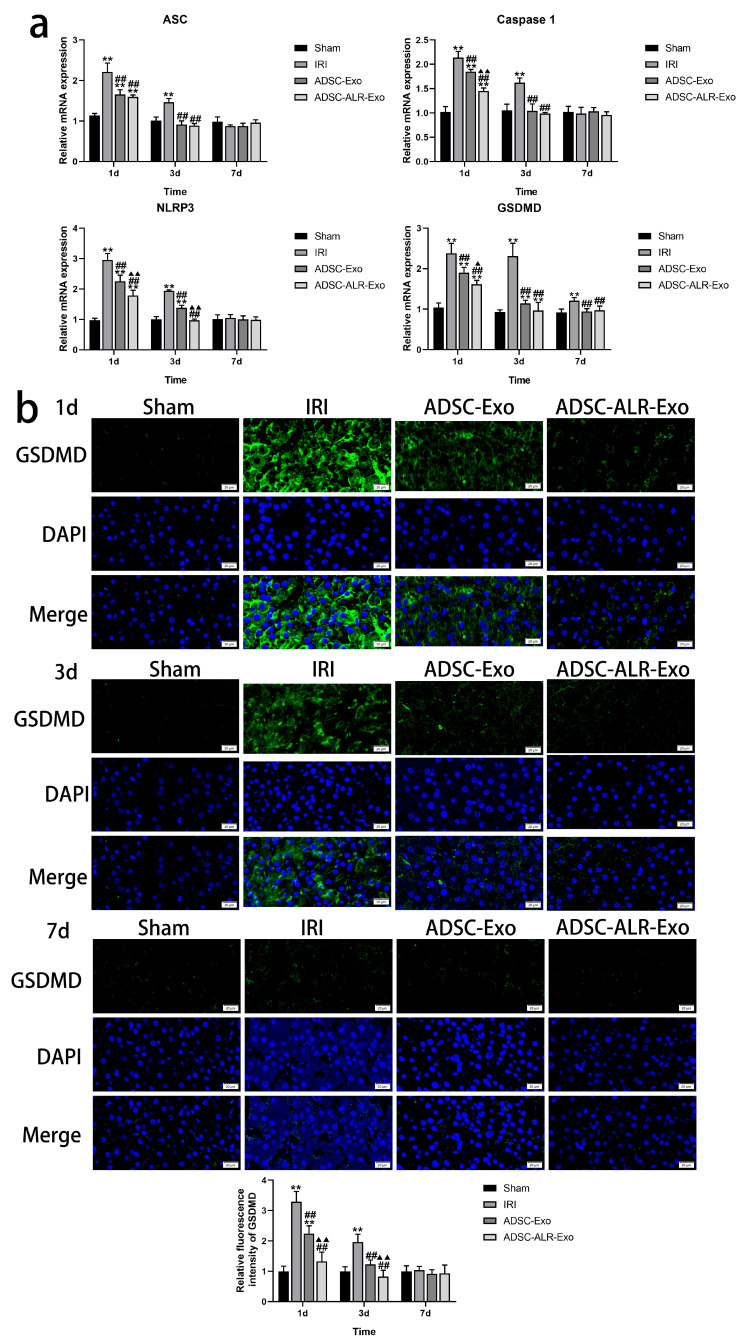
ADSC-ALR-Exo reduces pyroptosis induced by liver injury. (**a**) Expression levels of mRNAs related to indicators of pyroptosis in the liver. (**b**) GSDMD fluorescence was observed in the liver tissue. Scale bar: 20 μm. Quantification of the fraction of GSDMD using ImageJ. The results are presented as the means ± standard deviations (SDs). ** *p* < 0.01, compared with the Sham group. ## *p* < 0.01, compared with the IRI group. ▲ *p* < 0.05, ▲▲ *p* < 0.01, relative to the ADSC-Exo group.

Pyroptosis is an inflammation-associated form of cell death that is distinct from both apoptosis and necrotic cell death. Its occurrence typically involves the activation of the NLRP3 inflammasome, cleavage of GSDMD, and the release of inflammatory factors such as IL-1β and IL-18, which are closely linked to IRI [39]. Our findings (Figure 5a) demonstrate that at one day post-surgery, the postoperative groups exhibited a significantly increased expression of pyroptosis-related genes, including *NLRP3*, *ASC*, *Caspase 1*, and *GSDMD*, compared to the Sham group (*p* < 0.01). Intervention with ADSC-Exo and ADSC-ALR-Exo substantially attenuated this increase (*p* < 0.01), with ADSC-ALR-Exo showing superior suppression effects, particularly for *NLRP3*, *Caspase 1*, and *GSDMD* mRNA expression (*p* < 0.05). By 3 days post-surgery, although the expression levels of all detected genes decreased in the IRI group, they remained significantly higher than those in the Sham group (*p* < 0.01). Notably, *NLRP3* and *GSDMD* mRNA expression in the ADSC-Exo group, as well as *GSDMD* mRNA expression in the ADSC-ALR-Exo group, still showed significant differences compared to the Sham group (*p* < 0.05). The results indicate that, compared to ADSC-Exo, the intervention with ADSC-ALR-Exo resulted in a downward trend in *ASC* gene expression, although this change was not statistically significant. We speculate that this may be due to the timing of *ASC* expression changes, which could occur either earlier or later than the time points we sampled. Alternatively, *ASC* may exhibit reduced sensitivity to ALR overexpression in ADSC-ALR-Exo, while other pyroptosis-related genes, such as *NLRP3*, *Caspase-1*, and *GSDMD*, may receive stronger inhibitory signals or be directly targeted, leading to decreased mRNA levels. By postoperative day 7, only *GSDMD* remained elevated in the IRI group (*p* < 0.01), while the other genes returned to normalized levels (*p* > 0.05). To further investigate the effects of ADSC-Exo and ADSC-ALR-Exo on cell pyroptosis following liver injury, we conducted immunofluorescence analysis of the pyroptosis substrate GSDMD. The results were consistent with the gene expression data (Figure 5b). At 1 day and 3 days post-surgery, GSDMD protein expression was significantly elevated in the IRI group (*p* < 0.01). The interventions with ADSC-Exo and ADSC-ALR-Exo significantly reduced GSDMD expression (*p* < 0.01), with ADSC-ALR-Exo demonstrating a more pronounced effect (*p* < 0.01). By postoperative day 7, no significant differences were observed (*p* > 0.05). These findings indicate that ADSC-ALR-Exo reduces NLRP3 inflammasome formation and further decreases the expression of GSDMD, the executor of pyroptosis.

## 4. Discussion

Hepatic resection has become an increasingly prevalent and highly regarded technique in contemporary liver surgery. This minimally invasive procedure offers numerous advantages for treating liver diseases, characterized by reduced trauma, accelerated recovery, and enhanced surgical visibility for surgeons [40]. However, hepatic resection inevitably exposes the liver to IRI, which often results in significant hepatic dysfunction and subsequently endangers patient survival [41]. Gaining a deep understanding of the pathogenesis of hepatic IRI and exploring effective prevention and treatment strategies are of significant clinical importance for improving patient outcomes and reducing medical risks. The anatomical structure and physiological functions of the liver in *miniature* *pigs* closely resemble those of the human liver, making them an ideal model for surgeons to conduct simulated training in liver surgery.

ADSCs have demonstrated significant biological activity in promoting wound healing, suppressing inflammatory responses, alleviating IRI, and improving liver function [42,43]. Research on ADSCs in tissue regeneration and injury repair has garnered extensive attention. Among numerous ADSC studies, exosomes have shown great potential for clinical application due to their high biocompatibility and low immunogenicity, which enable them to function as intercellular signaling carriers [44]. In a model of neuronal injury following cardiopulmonary resuscitation (CPR), exosomes derived from CXCR4-modified MSCs effectively suppressed pyroptosis, repaired neuronal damage, and enhanced behavioral recovery [45]. Furthermore, existing studies have demonstrated that bone morphogenetic protein 2 (BMP2)-modified MSC-Exo effectively promotes bone regeneration and accelerates bone healing [46]. Genetic engineering techniques are employed to modify and engineer exosomes, endowing them with more targeted therapeutic effects. These genetically modified MSC-Exo exhibit broad application prospects in the biomedical field. As a novel hepatocyte growth factor, the ALR specifically stimulates liver regeneration following partial hepatectomy, facilitating the treatment and repair of liver injury and disease [47]. Therefore, this study infected ADSCs with ALR via an adenoviral vector to obtain ALR-rich ADSC-Exo for intervention in a liver IRI combined with partial hepatectomy injury model [30]. This study demonstrates that ADSC-Exo overexpressing ALR effectively mitigates ultrastructural alterations induced by hepatic IRI, suppresses oxidative stress and inflammatory responses, and reduces endoplasmic reticulum stress and pyroptotic damage. Compared to conventional exosomes, ADSC-ALR-Exo exhibits superior therapeutic efficacy, further validating the potential of ADSC-Exo as a gene delivery vehicle.

Following IRI, alterations in the hepatic microenvironment impact the antioxidant system within hepatocytes, diminishing their ability to scavenge free radicals and resulting in persistent oxidative damage. After hepatic IRI, excessive inflammatory responses exacerbate hepatocyte injury and impede tissue repair [32,33]. Our findings indicate that following hepatic IRI, ROS levels in liver tissue increase, antioxidant enzyme activity is inhibited, and levels of the lipid peroxidation product MDA rise, leading to oxidative damage. Concurrently, levels of pro-inflammatory factors in both blood and liver tissue increase, while levels of anti-inflammatory factors decrease, resulting in the activation of an inflammatory response. Clinical studies on liver injury indicate that ALR protects hepatocytes from nonalcoholic fatty liver disease (NAFLD) by regulating mitochondrial function, suppressing oxidative stress responses, and promoting hepatocyte regeneration. A deficiency of ALR in hepatocytes induces oxidative stress in liver tissue, leading to impaired hepatocyte function and resulting in rapid apoptosis or necrosis [48,49]. Furthermore, ALR deficiency accelerates the progression of NAFLD from simple steatosis to nonalcoholic steatohepatitis, liver fibrosis, and potentially cirrhosis [50]. In studies of hepatic IRI, ALR intervention was shown to suppress Kupffer cell activation and neutrophil chemotaxis, reduce pro-inflammatory cytokine production, further diminish inflammatory responses, decrease apoptosis, and promote liver function recovery [51]. TS Weiss et al. also demonstrated that ALR effectively reduces inflammation and oxidative stress induced by hepatic IRI, thereby mitigating liver injury [52]. Our previous study found that ADSC-Exo exhibited therapeutic effects in alleviating oxidative stress and systemic inflammatory responses caused by hepatic IRI combined with partial hepatectomy [31,53]. In our study, the incorporation of the ALR gene significantly enhanced the anti-inflammatory and antioxidant capabilities of ADSC-Exo in the context of hepatic IRI. This enhancement was characterized by a reduction in the release of inflammatory factors, a decrease in the levels of lipid peroxidation products and ROS, and an increase in the activity of antioxidant enzymes.

In recent years, the role of ER stress and its associated signaling pathways in hepatic ischemia–reperfusion injury has garnered increasing attention. Multiple experimental studies in liver I/R injury models have demonstrated that ER stress markers, such as GRP78/BiP, CHOP/GADD153, phospho-eIF2α, spliced XBP1, and ATF6 activation, are significantly upregulated following IRI [54,55]. This activation is typically observed in the early stages of reperfusion and correlates positively with the extent of hepatocyte injury, oxidative stress, inflammatory response, and apoptosis rates [56,57,58]. Consistent with our findings, this study also demonstrated that following hepatic IRI, there is an increased translocation of the ER lumen molecular chaperone GRP78/BiP. The activation of ER stress-related signaling pathways, including PERK, IRE1, and ATF6, leads to the onset of ER stress. Consequently, the ultrastructure of the hepatic ER is disrupted, resulting in severe damage to hepatocytes. This damage may be associated with the oxidative stress and inflammatory response induced by hepatic IRI. ALR plays a crucial role in maintaining cellular homeostasis, promoting liver regeneration, and regulating redox balance. Increasing evidence indicates that ALR influences endoplasmic reticulum stress through multiple pathways. Xiao et al. demonstrated that ALR effectively suppresses ER stress by modulating the interaction between BCL2 and IP3R, thereby reducing ER-Ca^2+^ release and mitochondrial Ca^2+^ uptake, which further mitigates PA-induced cellular damage [59]. Furthermore, studies on acute kidney injury have shown that overexpression of ALR effectively suppresses H_2_O_2_-induced ER stress responses, maintains endoplasmic reticulum morphology, and consequently reduces ER stress-mediated cell death in HK-2 cells [60]. In this regard, our findings indicate that the enrichment of ALR not only enhances the antioxidant capacity of ADSC-Exo but also significantly increases its inhibitory effect on ER stress induced by hepatic IRI, thereby alleviating damage to the ER ultrastructure and maintaining ER morphology. Consistent with our previous findings, ALR binding similarly enhances the anti-apoptotic capacity of ADSC-Exo and protects against mitochondrial structural and functional damage induced by hepatic IRI [30]. Based on this, we speculate that ALR-modified ADSC-Exo may possess ALR-related functions, and its therapeutic effects on ER stress-induced damage may be associated with its antioxidant effects, anti-apoptotic properties, and protection of mitochondria.

Pyroptosis is intricately associated with ER stress. In the classical pyroptosis pathway, GSDMD acts as the executor of pyroptosis, with its cleavage mediated by caspase-1. ER stress, as an upstream trigger, can activate inflammasomes through various molecular pathways, regulate caspase-1 expression, and potentially influence GSDMD localization and its pore-forming capacity by altering the membrane lipid environment [61,62,63]. Lebeaupin et al. demonstrated that ER stress activation in hepatocytes can initiate NLRP3 inflammasome activation, thereby inducing pyroptosis; conversely, ER stress inhibitors were shown to mitigate this effect [64]. Our investigations into hepatic IRI revealed that ADSC-Exo also effectively suppressed pyroptotic injury [53]. Research has demonstrated that inhibiting pyroptosis significantly mitigates liver damage in an IRI model, a mechanism potentially linked to mitochondrial damage [65]. ALR exhibits notable antioxidant and anti-inflammatory effects in hepatic IRI. Gandhi et al. identified that ALR plays a crucial role in maintaining mitochondrial function in liver cells [66]. Specifically, ALR protects mitochondrial function from liver ischemia–reperfusion injury by inhibiting mitochondrial fission [67]. In this context, our study shows that, upon conjugation with ALR, ADSC-Exo more effectively reduces the formation of the NLRP3 inflammasome, subsequently decreasing the expression of the pyroptosis substrate GSDMD, reducing the release of pro-inflammatory cytokines IL-1β and IL-18, and inhibiting pyroptosis. Based on our previous hypotheses and the findings discussed, we propose that the therapeutic effect of ADSC-ALR-Exo on hepatic IRI may be associated with its inhibitory effects on pyroptosis and endoplasmic reticulum stress, suggesting a significant mechanistic link between these two processes. Further investigation is warranted to explore this relationship.

Our study has preliminarily demonstrated, through in vivo experiments, the protective effect of ADSC-ALR-Exo against liver injury induced by hepatic IRI. We hypothesize that there may be a causal relationship between its inhibitory effects on endoplasmic ER stress and pyroptosis; however, the specific mechanisms of action have yet to be validated through in vitro experiments. Therefore, our subsequent experiments will build upon this study by conducting in vitro experiments using hepatocytes or hepatocyte cell lines to investigate the mechanism by which ADSC-ALR-Exo modulates the relationship between ER stress and pyroptosis in the context of hepatocyte hypoxia-reoxygenation injury.

## 5. Conclusions

In summary, our study utilized *miniature pigs* as experimental animals for surgical modeling and conducted postoperative monitoring for up to one week to evaluate the therapeutic effects of ALR-modified ADSC-Exo. The results indicated that the combination of the ALR gene and ADSC-Exo exhibited synergistic effects in protecting the liver while alleviating the inflammatory response and oxidative damage associated with hepatic IRI. This therapeutic effect may be attributed to the effective suppression of ER stress and a reduction in pyroptosis. These findings underscore the therapeutic potential of ADSC-ALR-Exo in ameliorating liver injury.

## Figures and Tables

**Table 1:** PCR Primer sequences.

Gene	Forward Primer (5′-3′)	Reverse Primer (5′-3′)
*IL-1β*	CAAGGAAGTGATGGCTAACTACGG	AAGGTCCAGGTTTTGGGTGC
*IL-18*	GCTGAAAACGATGAAGACCTGG	CAAACACGGCTTGATGTCCCT
*IL-10*	GCATCCACTTCCCAACCAGC	CAGCAACAAGTCGCCCATCT
*IL-6*	CTTCAGTCCAGTCGCCTTCTCC	CATCACCTTTGGCATCTTCTTCC
*TNF-α*	CCACCACGCTCTTCTGCCTACT	CGACGGGCTTATCTGAGGTTTG
*GRP78*	TCGCATCCCAAAGATTCAACA	TCCCACGGTTTCAATACCAAGT
*ATF6*	CAGAGCCGCTAAAGGAAGATAAG	TGGAGTTTGTTTGAGTCTTGGGT
*IRE1α*	CTGAGCGAAGACTGCAAGGA	GAGTATGTTGGCCTGACGCT
*XBP1*	CCAGTTGTCACCCCTCCAGA	GGTCCAAGTTGAACAGAATGCC
*PERK*	ACCATCCGTTAAAATACGCAGAG	GCCACAGGAAATCCCCATAGA
*eIF2α*	ATTTGGCATTATACTGGCTCTGTCC	CGGTTTCTCATTTCCTGGTTGTG
*ATF4*	ATGGCCGAGATGAGCTTCCTGAGCA	TGCTCAGGAAGCTCATCTCGGCCAT
*JNK*	CGTGGATTTATGGTCTGTGGG	CGAATCGGCTGGGAAAAGTA
*CHOP*	AGGTGCTGTCCTCAGATGAAAATG	AGAGGCAGGGTCAAGAGTGGTG
*NLRP3*	CAGATGAACAGCAAGCAAGGGAA	CAGACCAGGGGAATAAAGCACA
*ASC*	AAAGCAGACAACAAACCAGCACT	TCCGTCAGCACCTTCCCGTA
*Caspase 1*	GCCATTAAGAAAGCCCACATAGA	ATAAGGGATGTCGCCAAGAAAC
*GSDMD*	GGACGCCATGCACCTTGAA	ACCTCCGTCACCACGAACAC
*β-actin*	TCTGGCACCACACCTTCT	TGATCTGGGTCATCTTCTCAC

## Data Availability

The original contributions presented in this study are included in the article/Supplementary Materials. Further inquiries can be directed to the corresponding author.

## Supplementary Materials

The following supporting information can be downloaded at: https://www.mdpi.com/article/10.3390/antiox15040450/s1, Figure S1: Original western blot images; Table S1: Gene accession number.

## Abbreviations

The following abbreviations are used in this manuscript:

ADSC-Exo	Adipose Mesenchymal Stem Cell-Derived Exosomes
ALR	Augmenter of Liver Regeneration
IRI	Ischemia–Reperfusion Injury
ER	Endoplasmic Reticulum
MSCs	Mesenchymal Stem Cells
