# Supplementary material for: Augmenter of Liver Regeneration-Modified Adipose Mesenchymal Stem Cell-Derived Exosomes Repairs Liver Damage by Regulating Endoplasmic Reticulum Stress and Pyroptosis in a Minipig Model of Liver Injury
Source: Antioxidants (Basel). 2026 Apr 3;15(4):450. doi: 10.3390/antiox15040450 (PMC13113251; doi:10.3390/antiox15040450)
Supplement: Supplementary file 1 [file antioxidants-15-00450-s001.zip › Table S1. Gene Accession Number.pdf]

Gene	Gene Accession Number
IL-1β	NM_214055.1
IL-18	NM_213997.1
IL-10	NM_214041.1
IL-6	NM_214399.1
TNF-α	NM_214022.1
GRP78	NM_001444629.1
ATF6	XM_021089510.1
IRE1α	XM_005668695.3
XBP1	NM_001271738.1
PERK	XM_003124925.4
eIF2α	XM_021069601.1
ATF4	NM_001123078.1
JNK	XM_003359272.4
CHOP	NM_001144845.1
NLRP3	NM_001256770.2
ASC	XM_003124468.5
Caspase 1	NM_214162.1
GSDMD	XM_021090506.1
β-actin	NM_001444420.1
