# Supplementary figures and images for: Augmenter of Liver Regeneration-Modified Adipose Mesenchymal Stem Cell-Derived Exosomes Repairs Liver Damage by Regulating Endoplasmic Reticulum Stress and Pyroptosis in a Minipig Model of Liver Injury
Source: Antioxidants (Basel). 2026 Apr 3;15(4):450. doi: 10.3390/antiox15040450 (PMC13113251; doi:10.3390/antiox15040450)

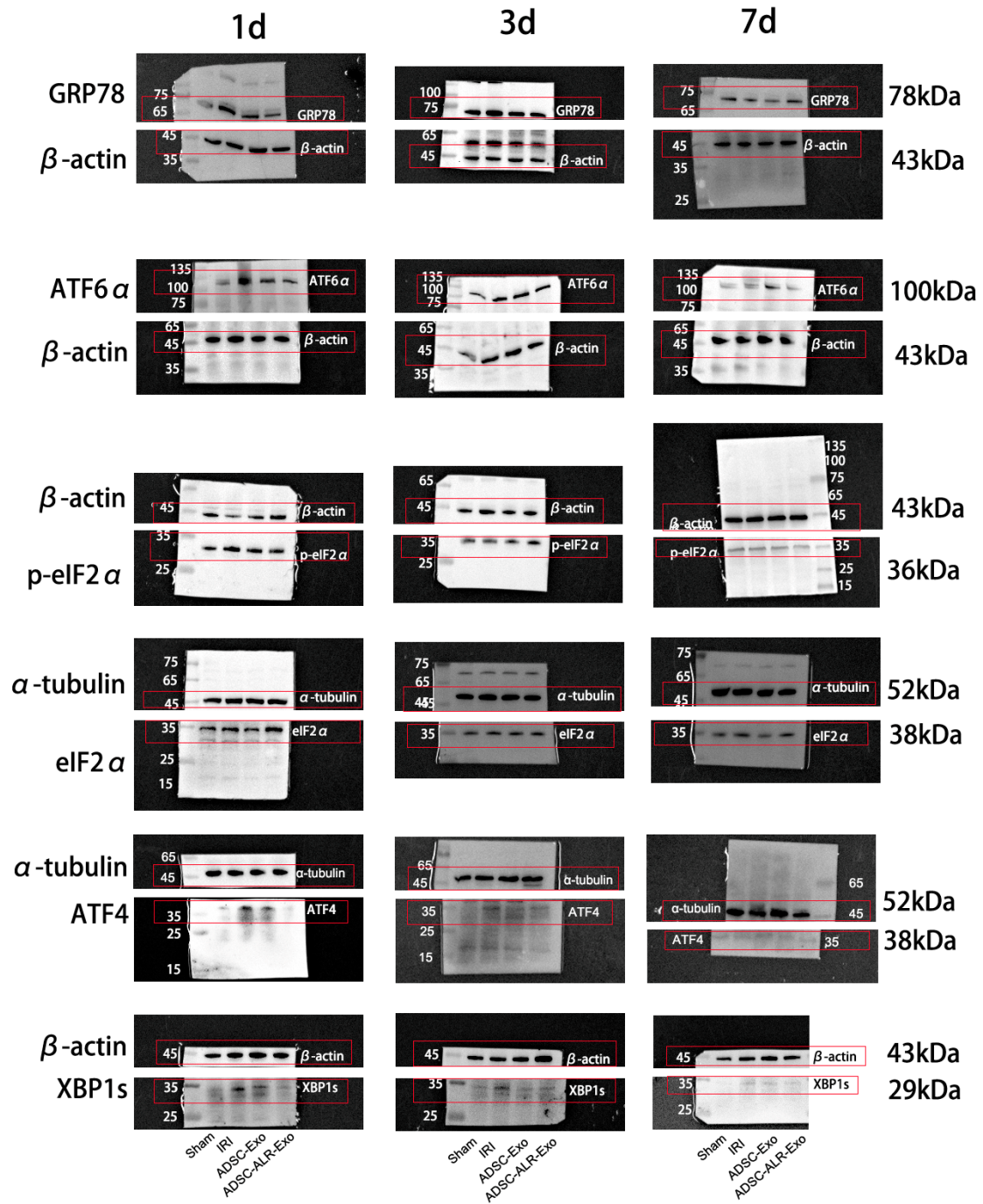

Supplement: Supplementary file 1 [file antioxidants-15-00450-s001.zip › Figure S1. Original Western Blots.pdf]
